# Simulation and mental health outcomes: a scoping review

**DOI:** 10.1186/s41077-016-0035-9

**Published:** 2017-01-28

**Authors:** Brett Williams, Priya Reddy, Stuart Marshall, Bronwyn Beovich, Lesley McKarney

**Affiliations:** 1 Department of Community Emergency Health & Paramedic Practice, Monash University, Peninsula Campus, PO Box 527, McMahons Road, Frankston, 3199 Victoria Australia; 2 Monash University, HealthPEER, Claytoria, Victoria Australia; 3 Latrobe University, Melbourne, Australia

**Keywords:** Students, Health occupations, Patient simulation, Mental health, Manikin

## Abstract

**Background:**

A scoping review was conducted in order to map and determine the gaps in literature on the impact of simulation as an educational approach to improve mental health care outcomes. As it became apparent that no literature existed on this topic, the study aimed to examine the educational impact of simulation on mental health education.

**Methods:**

An established five-stage scoping methodology was used: (1) identification of the research question, (2) identification of relevant studies, (3) study selection, (4) charting the data and (5) collation, summarising and reporting of results. CINAHL, ProQuest, PubMed, MEDLINE, EMBASE and PsychINFO databases were searched. These databases were deemed to represent a majority of the literature while accommodating for the particular search strategy used for this review. Websites that provide grey literature were also searched for articles of relevance.

**Results:**

A total of 48 articles were included in this review, with a considerable portion of studies conducted in the USA and UK. Others were conducted in an array of locations including Australia, Canada, Iran and Taiwan. Of the included articles, seven groups of simulation methods (including standardised patients, virtual reality and manikins as patients) were evident, with standardised patients being most prominent.

**Conclusions:**

Literature is lacking to evidence the benefit of simulation on mental health patient outcomes. However, the available literature suggests a variety of simulation-based education, and training methods are currently being used within mental healthcare education. The findings do suggest some methods of simulation, such as the use of standardised patients, are more commonly used in education and have been deemed as effective to assist in mental health education. As no article specifically examining the mental health outcomes of patients treated by health professionals taught by simulation was identified, the educational outcomes outlined in this paper may be used to inform further research, incorporating mental health patient outcomes.

**Electronic supplementary material:**

The online version of this article (doi:10.1186/s41077-016-0035-9) contains supplementary material, which is available to authorized users.

## Background

In healthcare education, simulation is used for both the teaching and assessment of students and staff. There are a variety of simulation-based education (SBE) and training methods, sometimes delivered in combination, dependent on the content and learning outcomes. The levels of difficulty, complexity and challenge can be tailored to suit the context, learning or assessment objectives and the experience level of the students [1]. The versatility of simulation allows it to take place in multiple settings including the classroom, clinical consulting room or hospital ward, simulation laboratory or the virtual world [2]. Many mental healthcare competencies are well-suited to SBE, and it can be used to expose students to clinical situations or events that occur infrequently or pose a high risk in terms of safety or liability [3]. However, some authors believe that simulation could be more widely adopted in the field of mental health education [4–6].

A preliminary literature search by the research team suggested very little information on the impact of simulation on mental health patient outcomes existed. Thus, a scoping study of the literature was performed to identify the extent of this literature gap and map what outcomes have been reported. At present, in the general medical literature, there are relatively few studies on the impact of simulation on patient outcomes and the collateral effects at a population level. This is especially true in the mental health sector [7, 8].

There is however significant research to show that simulation-based health education promotes knowledge acquisition and maintenance of clinical knowledge, attitude and skills [7, 9, 10]. A variety of simulation methodologies have been used in healthcare education including; actors trained to portray a person with a particular health concern (a simulated patient (SP)) [11–13], manikins and computer-generated scenarios [3]. These methods can range from “low-fidelity” where the level of realism is low to “high-fidelity” where there is a high degree of realism such as human patient simulators or manikins that are able to replicate a growing range of physiological signs [14].

Simulation offers many opportunities for the development of skills, knowledge and behaviours for students and clinicians working in mental health settings. Simulation also provides opportunities to address the challenges related to stigma, safety and liability present in the psychiatric clinical setting [3]. For people living with a mental illness, there are often compounding social, cultural, economic, family or other factors that may form part of their presentation or care. Well-trained and briefed simulated patients in well-designed simulation scenarios can portray the complexities of mental illness with high fidelity [15]. Simulation can help familiarise students with mental illnesses before they encounter them in a clinical setting, increasing the student’s ability to appropriately and confidently respond to patient needs.

The authors were unable to identify any literature on simulation as an educational approach affecting mental health patient outcomes. Therefore, this study was broadened to examine the educational impact of simulation on mental health education outcomes.

## Methods

Scoping reviews aim to identify the nature and extent of existing literature on a selected topic and can indicate the value of venturing into a full systematic review [16, 17]. A scoping review was chosen for this study as it had been identified in a preliminary literature search that little information existed in this area, and this methodology allowed for a broader body of literature which may underpin the topic to be investigated. Due to the apparent lack of pre-existing information, a systematic review was deemed as an unsuitable approach. With the use of Levac, Colquhoun and O’Brien’s [16] five-stage methodology for scoping reviews, a range of literature sources including grey literature sites were incorporated to complete a comprehensive review. These stages are as follows:

1. Identify the research question
2. Identify relevant studies
3. Study selection
4. Charting the data
5. Collating, summarising and reporting results

### Identify the research question

With the incidence of mental illness increasing globally, health occupations require innovative and improved mental health educational methods [18]. The breadth of literature available on the use of simulation in medical education has led to the proposal of the research question “Can simulation improve mental health outcomes?” The topic was deemed to be focused yet broad enough to conduct an effective scoping review to determine the gaps in the literature around the use of simulation and mental health outcomes. As our preliminary investigation into the literature identified a lack of information regarding patient outcomes, this broad approach also enabled information to be captured around related issues such as the types of simulations being used in mental health care, as well as skills and knowledge outcomes of students and clinicians. Due to the lack of literature identified examining patient outcomes, the research question evolved to examine educational outcomes as a platform to inform future patient outcome studies.

### Identify relevant studies

**Fig. 1:**
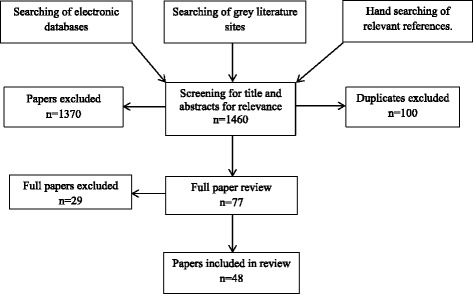
Search strategy methodology

A comprehensive literature search of six online peer-reviewed databases was conducted. These included CINAHL, ProQuest, PubMed, Ovid MEDLINE, EMBASE and PsychINFO. No restriction was placed on dates or locations of publications. The search strategy and results can be seen in Table 1 and Fig. 1. The full search strategies for each database are presented in Additional file 1. Three grey literature sites were also examined for non-peer-reviewed articles, these included http://www.greylit.org/, Google Scholar and http://www.tripdatabase.com/. Hand searches were also undertaken on full-text articles.

**Table 1:** Key search terms

	Main concepts		
	Simulation	Mental health	Health occupations
Search Terms	“mannequin” MH “Patient Simulation” MH “Simulations+” “simulation, medical” “Health Personnel as Patients+” “virtual patient” MH “Clinical Competence”	exp Mental Health/exp Mental Disorders/“mental conditions”	Health Occupations/Allied Health Personnel/ exp Primary Health Care/ exp Students, Health Occupations

“MH” refers to Mesh Heading and “exp” refers to Explode

### Study selection

To be included, articles were required to satisfy the three following inclusion criteria:

1. Articles incorporating mental health
2. Articles including simulation educational methods/outcomes
3. Articles relating to health professionals and/or health students.
  Articles were excluded if they were not written in English or did not meet the above inclusion criteria in terms of population, context or concept [19]. The search was conducted by one author and ratified by an expert librarian; screening and full-text reviews were completed by two authors. Consensus was reached on all full-text papers.

### Charting the data

Once the studies were selected, the spreadsheet program Excel was used to enter relevant information about each study such as intervention type, population studied and outcomes. This information was then synthesised by sorting and grouping the literature according to common themes. Analysis of the included articles determined several different simulation methods which are used in mental health education, and these interventions were examined to determine their effectiveness in improving student/clinician knowledge and skill development.

To assist with condensing the literature, the simulation methods were grouped based on key components; these included the following:

1. Simulation utilising people as patients
2. Simulation utilising virtual reality
3. Simulation utilising manikins as patients

### Classification of educational outcomes: Kirkpatrick’s model

The Kirkpatrick model is a framework created in order to determine the efficacy of a particular intervention, training or study [20]. It also allows educators, researchers and teachers to assess and objectively evaluate the effectiveness of the intervention [20]. It aims to guide the users on ways and areas of improvement based on participant involvement, achievement and growth [20, 21]. The model was utilised in this study as it has been widely cited in the educational literature and was developed to enable the evaluation of a variety of interventions in many environments [21–23]. Thus, it was deemed a more appropriate model for our purposes compared with others which were developed for use in more specific situations, such as within continuing medical education [24]. Furthermore, while Kirkpatrick’s model may not be useful in determining individual study quality, it is a useful tool to measure the progress of an emerging body of research aiming to eventually impact patient outcomes [25], such as SBE. Based on its means of utilisation, this model was included to further strengthen the review and assess the studies included. The levels, where level 4 is generally the most desired outcome for intervention, are as follows (Table 2):

**Table 2:** Four levels of Kirkpatrick’s model

Level 1 = participants react favourably to the learning or intervention.
Level 2 = participants acquired knowledge, skills and attitudes based on the intervention or study.
Level 3 = participants applied what they learnt into practice.
Level 4 = once applied, there was an outcome to that application of skills learnt from the intervention.

### Collating, summarising and reporting results

This stage of the scoping study involves organisation of the identified information to provide an overview of the existing literature on the topic. Thematic analysis of information from the included studies is performed to address the research question as well as to identify gaps in the literature.

## Results

This initial search yielded a total of 1460 articles from the online peer-reviewed databases (after eliminating duplicates) while the grey literature sites and hand searches produced no new results. A title and abstract screening located 77 articles that were to be included for the next stage in sorting. A further full article review yielded 48 results which are included in this review.

Among the 48 articles included for this review, 29 were conducted in the United States of America (USA), seven in the United Kingdom (UK), five in Australia, two in Canada and one each in the countries Taiwan, Italy, Malaysia, Iran and Germany. Furthermore, seven different types of simulation methods were identified, including the following:

- Simulated/standardised patients and actors [1, 11–13, 26–38]
- Virtual reality [6, 39–46]
- Role play [47–52]
- Manikins [14, 53–55]
- Computer simulation [56–59]
- Objective structured clinical examination (OSCE) [60–63]
- Voice simulation (which refers to the use of sounds and voice through an electronic medium to portray the sounds encountered by a schizophrenic patient) [18, 64–66]

From these articles, 39 suggested improvements in mental health education outcomes while the remaining nine articles either suggested no benefit or was used to assess clinician knowledge. Furthermore, 26 of the 48 articles focussed on undergraduate students, seven on postgraduates and 17 on clinicians. As some articles included more than one category of participant, the total is greater than 48. A full list of 48 included publications can be found in Additional file 2.

The Kirkpatrick model [20] assisted in providing strength to the review (see Additional file 2 for the Kirkpatrick ranking of each study). The lack of patient outcome data is evident in the results of this review, with the vast majority of articles scoring 2 or 3 on Kirkpatrick’s model. The current literature on mental health education using simulation lacks the patient outcome data and therefore provides abundant opportunities for future research and evaluation.

## Discussion

Education for mental healthcare is an important topic as there is a high incidence of mental illness worldwide, and it is projected to be an increasing cause of burden of disease in the future [67, 68]. Research and technology is constantly improving, as such the understanding of mental illness is also improving, creating new treatment and education methods for clinicians and healthcare students [69]. SBE has been viewed as an educational method with the potential to improve the care of individuals with mental illness [49]. The use of simulation has been implemented in medical and nursing education for decades with evidence suggesting its benefits, to both learners and patients [52].

This current scoping review was conducted in order to determine the existing evidence regarding the use of simulation and its effect in improving mental health patient outcomes. However, the scoping review did not uncover any literature reporting on patient mental health outcomes related to SBE. As a consequence of the broad literature search strategy, current information was also gathered on other outcomes of SBE within mental health such as learner reactions to SBE (Kirkpatrick model, level 1), knowledge and educational outcomes (Kirkpatrick model, level 2) and application of this education (Kirkpatrick model, level 3). In an environment where no patient outcomes have been reported, knowledge, skill enhancement and skill application are important outcomes which may inform future studies of SBE in relation to patient mental health outcomes.

The seven simulation types identified by the scoping review were SPs (*n* = 17), virtual reality (*n* = 9), role play (*n* = 6), manikins as patients (*n* = 4), computer simulation (*n* = 4), OCSE (*n* = 4) and voice simulation (*n* = 4). These results suggest that although SBE is being used, it has received very little empirical examination in the mental health sector. The following discussion will focus on the skill enhancement and efficacy of various forms of simulation used in mental health education, and potential links to mental health patient outcomes.

### Simulated standardised patients (SPs)

The use of individuals to portray patients has been suggested as the most effective way to educate healthcare professionals in communication and other clinical skills [13]. Communication skills are vital for any healthcare workers with the need for developing effective inter-personal techniques a central component in all patient/client interactions, especially within mental healthcare [70]. SPs are a valuable modality when there is a high degree of emotion and/or communication required, as they can provide non-verbal as well as verbal information and responses [1].

#### SPs: skill enhancement

Hall [12] noted that the use of actors to portray psychiatric patients was effective in enhancing the assessment and therapeutic communication skills of nursing students. Of the 112 students that were a part of the pilot study, 80% agreed that the SP accurately portrayed depression and 100% of the cohort reflected improved communication [12].

A mixed methods study by Lewy [28] reported that paediatric residents (*n* = 34) working with SPs attained an increased confidence in patient treatment, with 69% stating the intervention was “extremely helpful”. Shahabudin [30] trained medical students to act as patients presenting with various mental illnesses in order to assess the knowledge and diagnostic abilities of 42 general practitioners (GPs). The findings were grouped into three categories based on the performance of the doctors: group A—where 11.9% of the GPs informed the SPs of their anxiety diagnosis, group B—28.6% of the GPs prescribed medication for anxiety but did not inform the SP of their diagnosis and group C—where 59.5% of GPs did not diagnose nor treat the SP [30]. This study highlighted the lack of consistent training, assessment and treatment in the GP management of mental health and highlights an opportunity for future research and investigation.

#### SPs: simulation efficacy

Fussell’s study in [38] suggested that actors portraying people with substance abuse provided a reliable and effective learning modality in the education and assessment of substance abuse counsellors. Role play was suggested by Roberts’ [50] randomised controlled trial to be neither effective nor ineffective in improving the assessment skills and views of undergraduate medical students regarding people living with a mental illness.

### Virtual reality (VR)

Virtual reality is a computer-generated scenario or environment with which an individual can actively interact [43]. The concept is becoming increasingly common in healthcare education with the concomitant decrease in risk to patient safety [71]. VR technologies can include the computer-generated scenarios of virtual environments, voice simulation and virtual patients. Seventeen articles included the use of virtual reality technologies with occupations incorporating a mix of nursing, medical and psychiatric students and practitioners. The general consensus between the main findings of the studies is that these methods are effective in mental health education [6, 41, 56].

#### VR: simulation efficacy

Guise [6] found that virtual patients can be very effective in teaching clinical decision-making skills to nursing students, especially those parts of distance learning groups. The reduced risk of negative consequences for incorrect diagnosis and treatment-assisted students in learning mental health clinical skills. Lambert [41] found that the use of a simulated virtual patient, or avatar, portraying a person living with a mental illness was effective in educating nursing students on appropriate communication methods. The study investigated 85 mental health nursing students who followed the in-hospital journey of the fictional avatar for a 2-week period. Although the study did not focus on any form of assessment, it found that the students became more understanding and ethical practitioners at the completion of the fortnight and urged other organisations to follow suit in their training methods [41].

Voice simulation is effective in portraying the experience of schizophrenia, and as Weiland’s [66] qualitative study suggested, it is a valuable tool in increasing patience and empathy in nursing students. Seventy-four students listened to audio recordings of common voices heard in schizophrenia while attempting to complete certain tasks such as a job application. Based on the reflective evaluations completed by the students post-intervention, common themes emerged included feelings of “frustration” and “feeling overwhelmed”. However, the experience had positive outcomes, with reporting of increased levels of patience and empathy towards schizophrenic patients [66].

#### VR: skill enhancement

Satter’s [44] study of 14 practicing primary care physicians (PCPs) found that they were slightly better at diagnosing major depressive disorder and post-traumatic stress disorder with the use of avatars, as compared to those who used paper-based scenarios. In another study, Heiser [56] found that psychiatrists had the same chance of correctly diagnosing paranoia for a computer-simulated patient or a real patient, which suggests that this method may be suitable for use in mental healthcare education. Given the technology capability in 1979, Heiser’s results may not be necessarily comparable with modern day technology but the general method of simulation used is still relevant and transferable today. VR is proving to be a more prominent method for mental health education in today’s society, particularly as technology advances. Its effectiveness is yet to be fully determined; however, the indication from the majority of studies is that VR is a positive way to educate healthcare professionals regarding mental illness.

### Manikins as patients

This scoping review only located four articles that utilised manikins in the mental health setting. All of these articles focussed on nursing students, suggested a positive response and portrayed the effectiveness of the use of manikins in mental health education.

#### Skill enhancement

A study by Kameg [54] found that students who were previously at risk of failing were no longer at risk after completing training with the use of high-fidelity manikins. The quasi-experimental study used a cohort of 35 nursing students to complete a 30-item Health Education Systems Incorporated (HESI™) custom exam pre- and post-simulation [54]. There was a statistically significant improvement in the student risk profile post-simulation, with 10 of the 13 “at risk” students improving their category to the “non-risk” level [54].

In an earlier study, Kameg [14] noted that the use of manikins was an effective approach in teaching communication skills to nursing students. Similarly, Grant’s [53] review paper noted that high-fidelity manikins, when in combination with OSCE’s, were a viable training source to improve therapeutic communication skills. Unsworth’s study in [55] utilised the SimMan (a medium-fidelity manikin) and found that the use of manikins demonstrated to students where their areas of weaknesses were and what needed to be improved for their prospective healthcare careers. The qualitative measures of the study revealed the students thought of the intervention as “bridging the gap” between developing vital skills that are rarely seen in practice but are necessary to understand [55].

### Mental health patient outcomes

Although much evidence exists to support the use of SBE in medical and nursing education, both in terms of educational and patient outcomes, there is far less evidence regarding its use in the healthcare category of mental health. Moreover, to the best knowledge of the authors, no studies have been published that report on patient mental health outcomes in relation to SBE. Many of the attitudes, skills and competencies necessary for effective mental healthcare are well-suited to SBE, and there appears to be abundant opportunities for the development and evaluation of this teaching methodology within mental health [3]. However, the mental health educational outcomes reported in this paper including knowledge, skill enhancement and skill application have relevance and may be used to inform future studies in the area, including those concerned with patient outcomes. The measurable patient outcomes in mental health interventions may be less tangible compared with that of physical disorders. This may present one of the challenges in taking the evaluation of simulation in mental healthcare beyond educational outcomes.

### Limitations

Articles not in English were excluded from this review; this is an important limitation and publication-bias as significant data may have been missed due to the inability to appropriately categorise the articles. The search of grey literature sites yielded only peer-reviewed articles; this suggests the depth of included articles does not incorporate non-peer-reviewed literature which could have brought strength to the findings. Also, with only three articles reporting on impacts in non-westernised countries [30, 32, 42], care must be taken when generalising results to the wider population.

### Recommendations for future research

The most significant gap in the current research base is the lack of evidence that mental health SBE directly impacts patient outcomes. This scoping review has found several gaps in the current literature that may provide researchers, policymakers and educators with a “roadmap” of future research opportunities. These include interdisciplinary research, patient outcomes, different methods of simulation (such as pre-recorded DVDs which may use simulated patients portraying various clinical situations), prehospital care and SPs.

Although there are a number of articles in the literature reporting on simulation in mental healthcare education, there is a lack of patient outcome measures linked to SBE. This was supported in the evaluation using Kirkpatrick’s model as nil level 4 studies were located in any of the 48 articles. Further research needs to be conducted into how simulation in mental healthcare relates to patients’ outcomes in terms of successful or unsuccessful treatment, including the quality of life of the patient and their family. It is the authors’ view that further research is also needed for professions other than medicine and nursing. As a large portion of the existing research focussed on these professions, the findings may not be generalisable to other disciplines. In addition, more focus needs to be given to out-of-hospital care, including emergency paramedic clinicians and GPs working with people experiencing acute mental health issues. A significant gap in the mental health literature relates to indigenous populations including Aboriginal Australians. Similarly, there is a dearth of literature on the use of SBE in the mental healthcare of paediatric patients, young adults and the elderly population.

The evidence suggests that robust evaluation of simulation programs needs to be undertaken to provide evidence of the impact of simulation in mental healthcare education beyond educational outcomes. SBE holds many opportunities for curricula improvement and development. Ideally, evaluation plans would be incorporated at the design phase of new programs and introduced into programs which already exist. That is, planning backwards and teaching forward, that consider Kirkpatrick’s model. Further research should consider and focus on designs that are both qualitative and quantitative to obtain both narrative and objective data. Consideration should also be given to improve reporting where SP and role play approaches are involved [72].

## Conclusions

Although SBE has been demonstrated to be beneficial in many aspects of healthcare education, to the best of the authors’ knowledge, there is no existing evidence regarding the effect of SBE on mental health patient outcomes. The literature suggests a variety of simulation methods are currently being used within mental healthcare education. As evidenced by the number of Kirkpatrick level 2 and 3 findings in research papers included in this scoping study, simulation has proved generally beneficial in terms of educational and clinical skill outcomes. However, the results remain variable and therefore not necessarily generalisable. Many attitudes, skills and competencies vital to mental healthcare practice are seen as well-suited to SBE methodologies. Therefore, further research would be valuable to comprehensively examine the effects of SBE, including that of patient outcomes. This research progress is necessary to add to the evidence base of mental healthcare.

## Additional files

Additional file 1: Search strategy for each database. (DOCX 15 kb)

Additional file 2: Data summary of publications. (DOCX 23 kb)
