# Supplementary material for: Simulation and mental health outcomes: a scoping review
Source: Adv Simul (Lond). 2017 Jan 28;2:2. doi: 10.1186/s41077-016-0035-9 (PMC5806484; doi:10.1186/s41077-016-0035-9)
Supplement: Supplementary file 1 — Search strategy for each database. (DOCX 15 kb) [file 41077_2016_35_MOESM1_ESM.docx]

## Additional file 1: Search strategy for each database

|  | **CINAHL** | **ProQuest** | **PubMed** | **MEDLINE** | **EMBASE** | **Psych Info** |
| --- | --- | --- | --- | --- | --- | --- |
| **Strategy** | (all(MH “Health Occupations+”) OR (MH “Students, Health Occupations+”) OR (MH Allied Health Professions+”) OR (“Health Personnel”) OR (MH “Allied Health Personnel”) OR (MH “Primary Health Care”) OR (MH “Rural Health Personnel”)) AND (all(MH “Models, Anatomic+”) OR “man*ikin” OR “mannequin” OR (MH “Patient Simulation”) OR (MH “Simulations+”) OR “simulation, medical” OR “Health Personnel as Patients+”) OR “virtual patient” OR (MH “Clinical Competence)) AND (all(MH “Mental Health”) OR (MH “Mental Disorders, Chronic”) OR (MH “Mental Disorders”) OR “mental illness” | (all(exp *Allied Health Occupations/) OR all((exp *Primary Health Care/ OR exp *Health Occupations/ )) OR all(student*, health occupation*)) AND (all(patient simulation*) OR all((mannequin* OR man*ikin*)) OR all((models, anatomic OR virtual* patient*))) AND (all(exp Mental Health/) OR all(mental disorder*)) | ((((student*, health occupation*) OR (((exp *Primary Health Care/) OR exp *Health Occupations/) OR exp *Allied Health Occupations/))) AND ((mental disorder*) OR mental health)) AND ((simulat*) OR (((((patient simulation*) OR mannequin*) OR mannikin*) OR manikin*) OR virtual patient*)) | ((mannequin*mp.) OR (OSCE.mp.) OR (manikin*mp) OR (man*ikin.mp) OR (computer*adj3 interact*) OR (computer*adj3 therap*) OR (clinical skill* adj3 assessment) OR (role adj3 play*) or (patient simulation.mp) OR (simulat*) OR (virtual adj3 patient*)) AND ((exp Mental Health/) OR (exp Mental Disorders/)) AND ((exp Health Occupations/) OR (Allied Health Personnel/) OR (exp Primary Health Care/) OR (exp Students, Health Occupations) | ((mannequin*mp.) OR (OSCE.mp.) OR (manikin*mp) OR (man*ikin.mp) OR (computer*adj3 interact*) OR (computer*adj3 therap*) OR (clinical skill* adj3 assessment) OR (haptic*) OR (anatomic model/) OR (patient simulation/)) AND ((exp Mental Health/) OR (exp Mental Disease/)) AND ((health occupations.mp) OR (medical profession/) OR (health care personnel/) OR (paramedical personnel/) OR (allied health professional.mp) OR (primary health care/) | ((mannequin*mp.) OR (OSCE.mp.) OR (manikin*mp) OR (man*ikin.mp) OR (computer*adj3 interact*) OR (computer*adj3 therap*) OR (clinical skill* adj3 assessment) OR (haptic*) OR (anatomic model/) OR (role adj3 play*) or (patient simulation/)) AND ((exp Mental Health/) OR (exp Mental Disorders/) OR (mental conditions)) AND ((health occupations.mp) OR (medical profession/) OR (health care personnel/) OR (Allied Health Personnel) OR (exp Primary Health Care/) OR (students, health occupations) OR (Medical Personnel) |
