# Supplementary material for: Simulation and mental health outcomes: a scoping review
Source: Adv Simul (Lond). 2017 Jan 28;2:2. doi: 10.1186/s41077-016-0035-9 (PMC5806484; doi:10.1186/s41077-016-0035-9)
Supplement: Supplementary file 2 — Data summary of publications. (DOCX 23 kb) [file 41077_2016_35_MOESM2_ESM.docx]

## Additional file 2: Data summary of publications (n=48)

| **Type** | **Title** | **Findings** | **Date**  **Location** | **Participant Group** | **Kirkpatrick Level (1-4)** |
| --- | --- | --- | --- | --- | --- |
| **Virtual reality/patients** | Optimizing clinical training for the treatment of PTSD using virtual patients | Yes, using virtual reality patients undergoing virtual therapy improving interviewing abilities and communication | 2009, USA | Mental health clinicians | Level 2 |
|  | What can virtual patient simulation offer mental health nursing education? | Yes, suited to teach clinical decision making, especially for online learning | 2012, UK | Nursing students | Level 2 |
|  | Enhancing intellectual empathy: the lived experience of voice simulation | Yes, the use of voice simulation to depict mental disorders enhance student empathy | 2009, USA | Undergraduate nursing students | Level 3 |
|  | Meet Mohammed: using simulation and technology to support learning | Yes, the use of the avatar (Mohammed) to practice clinical communication skills was effective | 2013, UK | Mental health nursing students | Level 2 |
|  | Effectiveness of a virtual patient program in a psychiatry clerkship | Yes, virtual patients are effective in providing supplemental training in clinical reasoning for psychiatry clerkship | 2012, Taiwan | Medical students | Level 3 |
|  | Virtual reality training for health-care professionals | Yes, can enhance learning process by increasing motivation and interest | 2003, Italy | Health care professionals | Level 2 |
|  | Avatar-based simulation in the evaluation of diagnosis and management of mental health disorders in primary care | Yes, those that used avatars were better at diagnosing PTSD and MDD then those who used paper based scenarios | 2012, USA | Primary care practitioners | Level 3 |
|  | A comparison of psychiatric decision-making by trainee general practitioners and trainee psychiatrists using a simulated consultation model (uses video-taped patients) | Looked at assessment and diagnostic abilities of the doctors | 1988, UK | General practitioners and psychiatrists | Level 2 |
|  | Virtual standardized patients: An interactive method to examine variation in depression care among primary care physicians | The use of interactive virtual patients for determining physician diagnosis of depression | 2008, USA | Primary care physicians | Level 1 |
| **Standardised patients/Actors** | Using children as simulated patients in communication training for residents and medical students: a pilot program | Yes, benefitted the students to improve assessment and interview skills working with child actors depicting ADHD and mental disorders | 2005, USA | Medical students | Level 2 |
|  | Making it real: Using Standardized Patients  to Bring Case Studies to Life | Yes, helped enhance and develop assessment skills and therapeutic communication skills | 2012, USA | Nursing students | Level 3 |
|  | Communication and patient safety in simulation for mental health nursing education | Yes, effectively teaches nurse patient communication using actors as standardised patients | 2012, USA | Undergraduate nursing students | Level 2 |
|  | Use of standardized patients to enhance a psychiatry clerkship | Yes, increased effectiveness in communicating and diagnosing five different mental illnesses using standardised patients | 2004, USA | Medical students | Level 3 |
|  | Use of actors as standardized psychiatric patients | Yes, actors played the role of grieving parents after the death of the child and staff benefited by improving skills | 2011, USA | Physicians, nurses, social workers, grief counsellors and administrators | Level 2 |
|  | Adolescent depression: evaluating pediatric residents' knowledge, confidence, and interpersonal skills using standardized patients | Use of actors to depict depression and drug abuse, believe that residents acted bias due to knowing they were being assessed therefore not a good tool to determine effectiveness | 2009, USA | First year residents | Level 1 |
|  | Simulations in drug training | Yes, gained useful information about patient feelings and behaviours which assisted | 1975, USA | Range of people from different background including five clinicians | Level 2 |
|  | Assessing the competence of general practitioners in diagnosing generalized anxiety disorder using standardized patients | Study was to understand GP knowledge on diagnosing anxiety using medical students as standardised patients | 1994, Malaysia | General practitioners | Level 1 |
|  | Standardized patients: a creative teaching strategy for psychiatric-mental health nurse practitioner students | Yes, increases cultural competency and assessment and communication skills | 2008, USA | Nursing practitioner students | Level 2 |
|  | Training and validation of standardized patients for unannounced assessment of physicians' management of depression | Psychology and nursing students were used as standardised patients to assess GP knowledge and assessment skill when being patients to unaware GP’s, did not look at GP skills. | 2009, Iran | General practitioners | Level 1 |
|  | Working together: a joint initiative between academics and clinicians to prepare undergraduate nursing students to work in mental health settings | Yes, it enhanced clinical skills through workshops that implemented mental health clinicians as actors | 2007, Australia | Undergraduate nursing students | Level 3 |
|  | End of third-year objective structured clinical examination: boon or bane? | Yes, using standardised patients for OSCE’s can improve general skill acquisition but not affective at all for psychiatric problem solving | 2008, USA | Medical students | Level 3 |
|  | Ratings of videotaped simulated patient interviews and four other methods of evaluating a psychiatry clerkship | Yes, after the 6 week program the students improved their interviewing skills | 1987, USA | Psychiatry clerkships | Level 3 |
|  | Portrayal of psychiatric disorders: are simulated patients authentic? | Yes, standardised patients are effective in teaching psychopathology but is not as effective as real patients | 2012, Germany | Psychiatrists | Level 1 |
|  | Promoting Therapeutic Communication and Patient-Centered Care Using Standardized Patients | Yes, improved safety and therapeutic communication | 2013, USA | Nursing students | Level 2 |
|  | An Evaluation of Mental Health Simulation with  Standardized Patients | Yes, it improved interviewing skills of patients with varied mental health issues | 2014, USA | Nursing Students | Level 2 |
|  | Evaluating and training substance abuse counselors: a pilot study assessing standardized patients as authentic clients | Yes, the use of actors as standardised patients are effective and reliable patient portrayals | 2009, USA | Substance abuse counsellors | Level 2 |
| **Manikins** | The impact of high fidelity human simulation on self-efficacy of communication skills | Yes, it is viable in communication training | 2010, USA | Nursing students | Level 2 |
|  | Simulation to enhance care of patients with psychiatric and behavioral issues: use in clinical settings | Yes, high-fidelity manikin and OSCE improve therapeutic communication skills | 2011, USA | Nursing students | Level 3 |
|  | Fusion of psychiatric and medical high-fidelity patient simulation scenarios: effect on nursing student knowledge, retention of knowledge, and perception | Yes, students who were previously at risk were now improved and not at risk after the training with high-fidelity manikins | 2013, USA | Nursing students | Level 3 |
|  | Recognition of physical deterioration in patients with mental health problems: the role of simulation in knowledge and skill development | Yes, increased confidence and highlighted areas of improvement for students | 2012, UK | Nursing students | Level 3 |
| **Role Play** | Automating individualized coaching and authentic role-play practice for brief intervention training | Yes, achieved higher scores after participating in online training and role play | 2010, USA | Medical and nursing students | Level 2 |
|  | Integrating a professional apprenticeship model with psychiatric clinical simulation | Yes, it enhanced communication abilities for nurses with schizophrenic patients through role play | 2011, USA | Undergraduate nursing students | Level 2 |
|  | Headspace theatre: an innovative method for experiential learning of psychiatric symptomatology using modified role-playing and improvisational theatre techniques | Yes, it was beneficial as it assisted knowledge/skill development for mental health and addiction | 2007, Canada | Medical students, psychiatric students, social workers, nurse, gambling therapists. | Level 3 |
|  | Effects of exposure to mental illness in role-play on undergraduate student attitudes | Role play did not improve nor did it diminish student attitude and perception towards mentally ill patients | 2008, UK | Medical students | Level 2 |
|  | Use of interactive teaching techniques to introduce mental health training to medical schools in a resource poor setting | Yes, after the use of role play MCQ improved and OSCE score in Somaliland | 2013, UK | Medical students and interns | Level 3 |
|  | A day in the life of an inpatient: an experiential game to promote empathy for individuals in a psychiatric hospital | Yes, it improved empathy and changed the way staff treated patients after the simulation games | 1990, USA | Psychiatric hospital staff | Level 2 |
| **Computer simulation** | Can psychiatrists distinguish a computer simulation of paranoia from the real thing? The limitations of Turing-like test as measures of the adequacy of simulations | Maybe, the computer simulation results in 5 accurate diagnosis and 5 inaccurate diagnoses | 1979, USA | Psychiatrists | Level 2 |
|  | Comparison of methods for teaching clinical skills in assessing and managing drug-seeking patients | No significant difference between exam results for students who were taught by standardised patients compared to computer | 2000, Australia | Medical students | Level 2 |
|  | 'eSimulation'. Part 1: Development of an interactive multimedia mental health education program for generalist nurses. | Yes, interactive online program allows for mass usage and encourages staff to make choices without fear of incorrectly diagnosing hence increasing confidence | 2013, Australia | Generalist nurses | Level 2 |
|  | 'eSimulation'. Part 2: Evaluation of an interactive multimedia mental health education program for generalist nurses | Yes, interactive online program allows for mass usage and encourages staff to make choices without fear of incorrectly diagnosing hence increasing confidence | 2014, Australia | Generalist nurses | Level 2 |
| **OSCE** | Validation of an objective structured clinical examination in psychiatry | Yes, this is a valid form of assessment, used eight stations in the OSCE | 1998, Canada | Psychiatry clerkship students | Level 2 |
|  | Psychiatry Clerkship Objective Structured Clinical Examination is Here to Stay | Yes, OSCE is an effective way to test communication skills but is expensive and should not be the sole assessment method | 2008, USA | Medical students | Level 2 |
|  | Teaching and assessing residents' skills in managing heroin addiction with objective structured clinical examinations (OSCEs) | Half-half, residents had better communication but not as good in management or assessment of risk behaviour in heroin abusing patients using standardised patients | 2013, USA | Medical residents | Level 2 |
|  | Teaching about Substance Abuse with Objective Structured Clinical Exams | Yes, students communication skills of substance abuse patients improved while confidence and interest increased | 2006, USA | Medical residents | Level 3 |
| **Voice Simulation** | Simulation in mental health education | Yes, improved confidence and attitude through the use of voice simulation through an MP3 player | 2011, Australia | Mental health professionals | Level 2 |
|  | Mindful Teaching Practice: Lessons Learned through a Hearing Voices Simulation | Yes, listening to MP3 while attempting to complete other tasks increased comfort with people who hear voices and increased patience and tolerance | 2015, USA | Mental health nursing students | Level 3 |
|  | Effects of simulated learning and facilitated debriefing on student understanding of mental illness | Yes, listening to sounds that may be heard by a schizophrenic for example increased empathy and patient understanding | 2010, UK | Occupational therapy students | Level 3 |
|  | Hearing distressing voices clinical simulation | Yes, hearing distressing voices and seeing hallucinations made students feel empathetic, understanding, patient and developed other ways of managing | 2014, USA | Nursing students | Level 3 |

**NB:** Kirkpatrick levels: 1 = Reaction, 2 = Learning, 3 = Behaviour, 4 = Results
1 = Participants react favourably to the learning or intervention.
2 = Participants acquired knowledge, skills and attitudes based on the intervention or study
3 = Participants applied what they learnt into practice
4 = Once applied there was an outcome to that application of skills learnt from the intervention (27)
